# Out of the Blue: Cyanosis and Methemoglobinemia in a Renal Transplant Patient Following Phenazopyridine Ingestion

**DOI:** 10.7759/cureus.94820

**Published:** 2025-10-17

**Authors:** Alexander M Osborne, Spencer Rice, Kyle Seifert, Andrew A Glickman, Darren Cohen

**Affiliations:** 1 Emergency Medicine, Nova Southeastern University Dr. Kiran C. Patel College of Osteopathic Medicine, Davie, USA; 2 Emergency Medicine, Memorial Healthcare, Hollywood, USA

**Keywords:** cyanosis, dyshemoglobinemia, emergency medicine, hypoxia, kidney transplantation, methemoglobinemia, methylene blue, nonprescription drugs, phenazopyridine, pulse oximetry

## Abstract

Methemoglobinemia is a rare but serious condition affecting the blood's ability to carry oxygen. Symptoms at presentation are often nonspecific. We describe the case of a 58-year-old female presenting with unexplained central cyanosis and severe hypoxia despite preserved respiratory effort and seemingly reassuring lab values. Further questioning revealed recent ingestion of a large quantity of phenazopyridine, an over-the-counter urinary analgesic known to cause oxidative stress. Co-oximetry confirmed significant elevation of methemoglobin levels. Methylene blue is well-regarded as the gold standard, and its use in treatment of this patient led to favorable clinical and laboratory improvement. Clinicians must maintain a high index of suspicion for dyshemoglobinemias in patients with unexplained cyanosis refractory to oxygen therapy, particularly when conventional metrics of oxygenation are inconsistent with the overall clinical picture.

## Introduction

Methemoglobinemia is a potentially life-threatening hemoglobin (Hb) disorder characterized by excess methemoglobin (MetHb) in the blood. MetHb forms when hemoglobin-bound iron is oxidized from its ferrous state (Fe²⁺) to its ferric state (Fe³⁺) [1]. MetHb is unable to bind oxygen and simultaneously increases the oxygen affinity of unaffected hemoglobin, which together reduces effective oxygen delivery to tissues [2]. MetHb is routinely generated as the body undergoes oxidative stress, and levels are effectively reduced by endogenous enzymes [1].

Acquired methemoglobinemia can be induced by certain medications such as local anesthetics, sulfonamides, nitrates, and phenazopyridine. Such medications can increase oxidative stress and overwhelm compensatory mechanisms [2,3]. While methemoglobinemia overall is uncommon, phenazopyridine-induced cases represent a clinically significant proportion of reported drug-related events [4-7]. A 2021 retrospective chart review analyzing over 1,000 methemoglobinemia cases from the National Poison Data System found that phenazopyridine accounted for 16% of drug-related cases, making it the second most commonly implicated agent. This likely reflects the drug’s widespread availability in both prescription and over-the-counter (OTC) forms [8].

Clinically, methemoglobinemia often presents with nonspecific symptoms, including headache, dyspnea, or fatigue. A more diagnostic feature is cyanosis unresponsive to supplemental oxygen in otherwise asymptomatic patients, sometimes referred to as ‘happy cyanosis’ [9-11]. Additional diagnostic clues arise from oxygenation assessment. Pulse oximetry often provides misleading oxygen saturation values. Arterial blood gas (ABG) analysis, measuring partial pressure of dissolved oxygen, may demonstrate oxygen levels that are within normal limits. Co-oximetry directly measures MetHb levels and is essential for accurate diagnosis [10].

## Case presentation

A 58-year-old female presented to the emergency department via EMS with a chief complaint of persistent heartburn. On arrival, vital signs were notable for oxygen saturation of 72% on room air, heart rate of 77 bpm, blood pressure of 122/79 mmHg, respiratory rate of 16 breaths per minute, and temperature of 37.2°C. Physical examination revealed central and peripheral cyanosis, with dusky discoloration of the lips and nail beds. Her past medical history included a cerebrovascular accident with residual left-sided weakness, remote epidural hematoma, hypertension, hyperlipidemia, depression, chronic headaches, and kidney transplant (2010). The patient was alert, speaking in full sentences, and in no apparent respiratory distress. She denied chest pain, cough, shortness of breath, or fever.

Initial laboratory workup included a room air ABG, which revealed a pH of 7.43, PaO₂ of 65 mmHg, SaO₂ of 93%, and a PCO₂ of 38 mmHg, consistent with evolving mild hypoxemia in the absence of hypercapnia. Additional laboratory findings are shown in Table 1. 

**Table 1:** Pertinent additional laboratory findings Abbreviations: urinalysis (UA), white blood cell (WBC), microliter (uL), nanograms per milliliter (ng/mL), millimoles per liter (mmol/L), high power field (/HPF).

Parameter	Patient Value	Unit	Reference Range
White blood cell	8.5	1000/uL	3.5 - 10.0
Troponin I	0.042	ng/mL	0.000 - 0.034
Lactate	1.0	mmol/L	0.7 - 2.0
UA, WBC	41 - 50	/HPF	0 - 3
UA, Nitrite	Positive	Negative	—
UA, Leukocyte esterase	2+	Negative	—
SARS-CoV-2 rapid test	Not detected	—	Not detected
Influenza rapid test	Not detected	—	Not detected

In response to the low oxygen saturation on presentation, the patient was initially trialed on non-invasive positive pressure ventilation (BiPAP), followed by high-flow nasal cannula (HFNC). Despite these interventions, oxygen saturation improved only modestly to 82%. Throughout this period, she remained without increased work of breathing or air hunger.

A CT pulmonary angiogram was obtained to evaluate for pulmonary embolism and was negative. Additional imaging, including a non-contrast CT of the brain and contrast-enhanced CT of the abdomen and pelvis, showed no evidence of any acute process. A chest X-ray was not performed during the initial workup.

**Figure 1:**
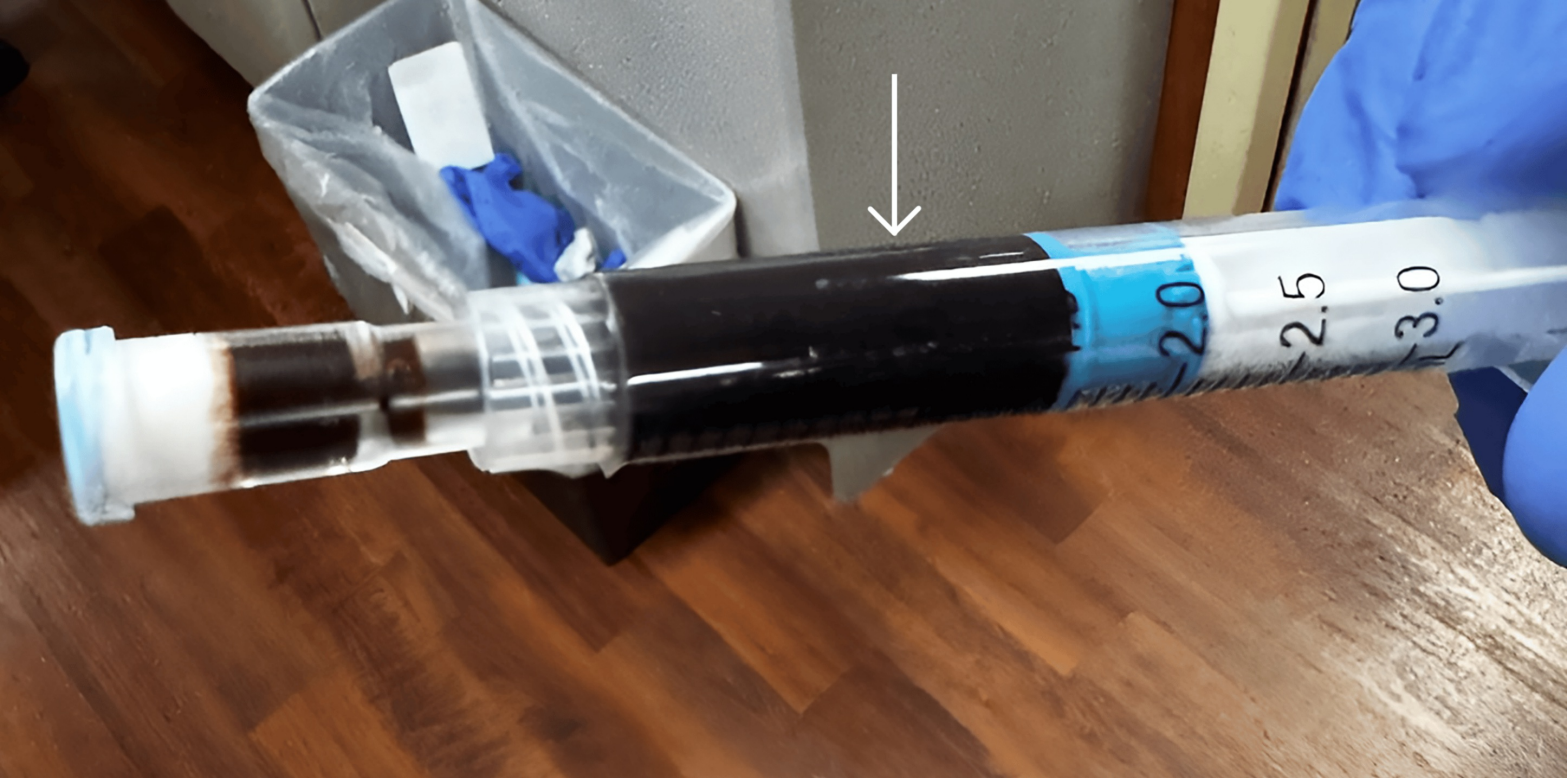
Arterial blood sample obtained in the emergency department, demonstrating a dark, chocolate-brown discoloration characteristic of methemoglobinemia. This abnormal hue results from oxidation of hemoglobin iron to the ferric (Fe3+) state, impairing its ability to bind and deliver oxygen.

A second ABG was obtained while on 100% FiO2 via HFNC, due to ongoing hypoxia and diagnostic uncertainty. Arterial blood appeared dark brown in color. Co-oximetry revealed a MetHb level exceeding 30%, with %O2Hb of 48.4% and a PaO2 of 316 mmHg (Figure 1). While the chocolate-brown discoloration was documented only on the second ABG, this characteristic finding was likely present earlier but not explicitly noted on the initial sample. These findings confirmed the diagnosis of methemoglobinemia (Table 2).

**Table 2:** Pertinent arterial blood gas values (timestamps included) Abbreviations: arterial blood gas (ABG), fraction of inspired oxygen (FiO₂), high-flow nasal cannula (HFNC), bicarbonate (HCO₃), methemoglobin (MetHb), oxyhemoglobin (O₂Hb), partial pressure of carbon dioxide (PCO₂), partial pressure of oxygen (PO₂), oxygen saturation (SO₂), total carbon dioxide (TCO₂), millimeters of mercury (mmHg), millimoles per liter (mmol/L)

Parameter	ABG #1 (13:42)	ABG #2 (15:04)	ABG #3 (17:00)	ABG #4 (05:00 next day)	Reference Range	Unit
pH, arterial	7.43	7.39	7.40	7.41	7.35 - 7.45	—
PCO₂	38	37	36	38	32 - 45	mmHg
PO₂	65	316	288	299	83 - 108	mmHg
HCO₃, arterial	25.2	22.4	22.3	24.1	21 - 28	mmol/L
TCO₂	26.4	23.5	23.4	25.3	19 - 24	mmol/L
%SO₂	93.0%	92.0%	92.3%	92.7%	94 - 100	%
%O₂Hb	—	48.4%	72.1%	84.7%	94 - 100	%
%MetHb	—	>30%	23.8%	9.2%	0.0 - 1.5	%
FiO₂	21%	100%	100%	100%	20 - 101	%
O₂ Device	Room Air	HFNC @ 60 L/min	HFNC @ 60 L/min	HFNC @ 60 L/min	—	—

Upon further questioning, the patient disclosed that she had ingested approximately 20 phenazopyridine (AZO) tablets the previous evening for urinary discomfort. Poison Control was consulted, and the patient was treated with 75 mg of intravenous methylene blue (MB) over 20 minutes at 15:55. A subsequent ABG (ABG #4 on Table 2) approximately one hour after MB administration showed improvement in oxyhemoglobin to 72.1% and a decline in methemoglobin level to 23.8%, indicating a favorable response to treatment. Oxygen saturation improved to 88%, and the patient remained hemodynamically stable. She was admitted to the intensive care unit for monitoring and continued to improve. The intensivist cited ABG #4 (Table 2) demonstrating high MetHb levels as the reason for initiating a second dose of MB at 03:30 the following day. Although the patient’s methemoglobinemia continued to improve, she remained in the hospital for antibiotic treatment for a urinary tract infection and continued immunosuppression due to her kidney transplant history. The patient's oxygen requirements were titrated down. MetHb levels progressively downtrended throughout the patient’s hospital course, and she was ultimately discharged on hospital day eight without complications.

## Discussion

**Figure 2:**
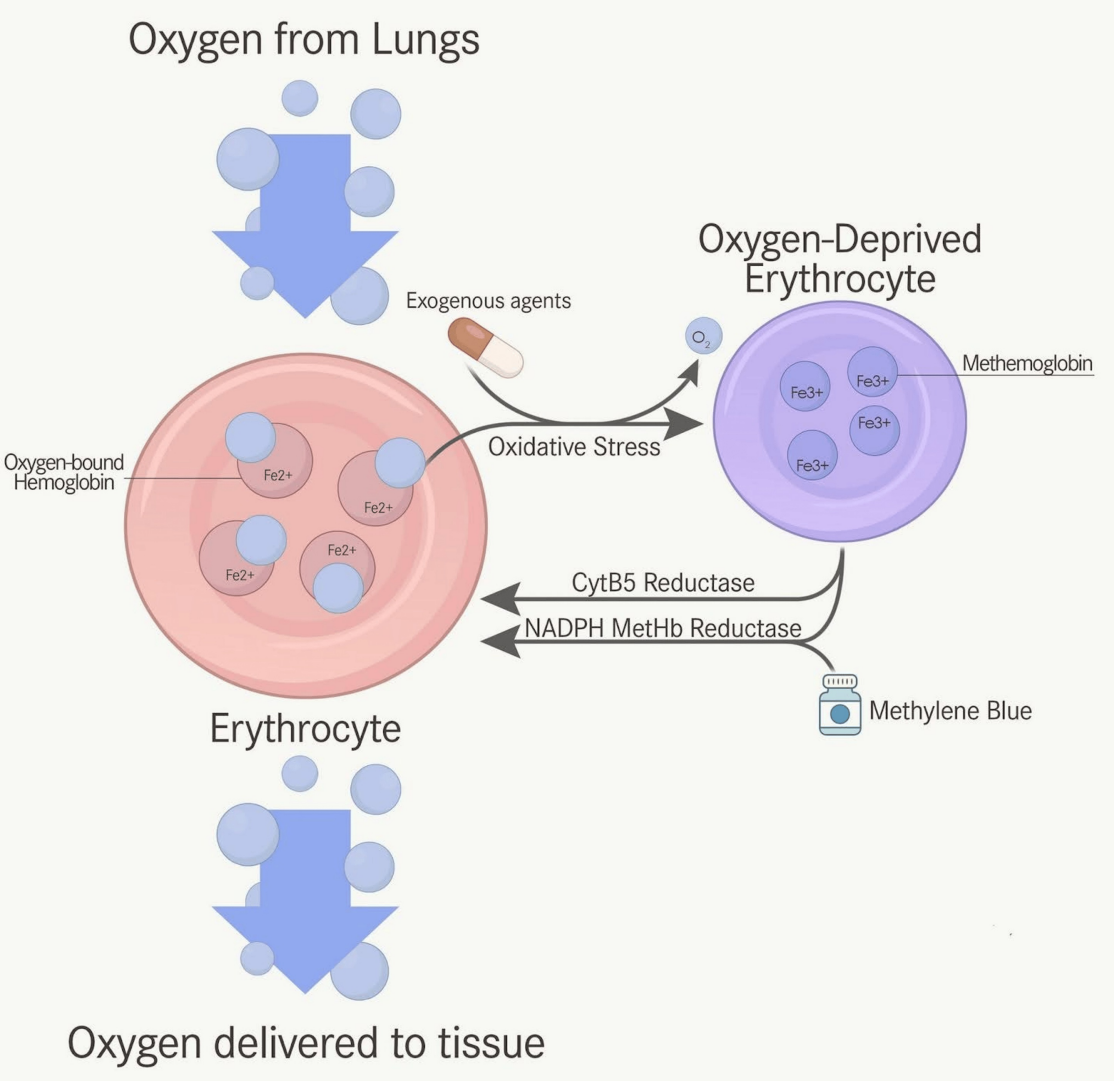
Oxygen binds to hemoglobin in the ferrous (Fe²⁺) state within erythrocytes for systemic tissue delivery. Exogenous agents such as phenazopyridine can exacerbate oxidative stress. This can convert Fe²⁺ to the ferric (Fe³⁺) state, producing methemoglobin (MetHb) and incapacitating its oxygen transport ability. The primary endogenous reduction mechanism involves the cytochrome b₅ reductase pathway, while the NADPH-dependent methemoglobin reductase pathway serves as an auxiliary route, pharmacologically enhanced by methylene blue. Figure created by the authors.

Methemoglobinemia is a rare but potentially life-threatening cause of hypoxia in the emergency setting. As shown in Figure 2, it results from the oxidation of hemoglobin’s iron moiety from the ferrous (Fe^2+^) to the ferric (Fe^3+^) state, which renders hemoglobin unable to bind and transport oxygen. In addition to this loss of oxygen-carrying capacity, MetHb also induces a leftward shift in the oxyhemoglobin dissociation curve, further impairing tissue oxygen delivery despite normal or elevated PaO2 values [1].

Phenazopyridine is a commonly available over-the-counter urinary analgesic, often perceived as benign by patients. However, its oxidative metabolites, particularly suspected aniline derivatives, can overwhelm endogenous enzymatic pathways such as cytochrome b5 reductase, leading to the accumulation of MetHb [12,13]. While phenazopyridine-induced methemoglobinemia has traditionally been considered rare [4-6], a 2021 study of over 1,000 methemoglobinemia cases reported to the National Poison Data System found that phenazopyridine accounted for 16% of drug-induced cases, second only to dapsone [8].

In this case, the patient presented with symptoms of heartburn and was found to have a positive urinalysis consistent with a urinary tract infection. She reported ingesting approximately 20 phenazopyridine tablets the night before presentation to relieve her symptoms. This underscores the importance of a detailed medication history, as patients often assume OTC medications to be harmless and may not initially report their use.

The clinical presentation was initially misleading. Despite an oxygen saturation of 72% on room air and visible cyanosis, the patient was alert, speaking in full sentences, and not in respiratory distress. Such a phenomenon has been described in methemoglobinemia as “chocolate cyanosis” or “happy hypoxia” [11]. Her initial ABG showed mild hypoxemia without hypercapnia, prompting escalation from nasal cannula to BiPAP, then HFNC, with little improvement. A negative CT pulmonary angiogram included in further workup did not identify a cardiopulmonary etiology.

Diagnostic clarity was achieved after co-oximetry revealed markedly elevated MetHb levels. On initial co-oximetry, %O2Hb was 48.4% and PaO2 was 316 mmHg, confirming the dissociation between arterial oxygen tension and functional oxygen saturation in dyshemoglobinemia [13]. After treatment, repeat values showed a rise in %O2Hb to 72.1% with a still-supraphysiologic PaO2 of 288 mmHg, consistent with clinical improvement despite ongoing impairment in oxygen-carrying capacity. Standard ABG and pulse oximetry can significantly overestimate oxygenation in these cases.

Supportive care with supplemental oxygen is a key component of initial management in methemoglobinemia, despite its limited ability to reverse tissue hypoxia [14]. Approximately one hour after arrival, the patient was trialed on BiPAP, after which she remained on continuous HFNC at 100% FiO2 for the remainder of her treatment. Administering high-concentration oxygen helps maximize oxygen delivery by the remaining functional hemoglobin. Definitive therapy with intravenous methylene blue led to clinical improvement and a measurable decline in methemoglobin concentration. MB acts as an artificial electron acceptor for NADPH methemoglobin reductase, reducing methemoglobin back to functional hemoglobin [13]. For methemoglobinemia treatment, MB is given intravenously at a recommended dosage of 1-2 mg/kg in a 1% solution over a period of five to 30 minutes [15]. The 75 mg our patient received falls in line with this recommendation, as she weighed approximately 68 kg.

While MB is the first-line treatment for symptomatic or severe methemoglobinemia, it is contraindicated in specific patient populations [16]. In individuals with glucose-6-phosphate dehydrogenase deficiency, MB can trigger hemolysis due to a lack of NADPH, which is required for its reductive mechanism of action [1]. Additionally, MB is a monoamine oxidase inhibitor and has been associated with serotonin toxicity in patients receiving serotonergic agents [17]. Its use during pregnancy also warrants caution. Although considered teratogenic when used in early gestation via intra-amniotic administration, limited case reports have documented successful use in later stages of pregnancy without adverse fetal outcomes [18]. In pregnant patients, a risk-benefit analysis and shared decision-making approach should guide treatment. In patients with contraindications to methylene blue, or instances where methemoglobinemia remains refractory to its use, exchange transfusion, ascorbic acid, and hyperbaric oxygen therapy are alternative treatments. Exchange transfusion can quickly restore oxygen-binding capacity by replacing a patient’s MetHb-containing red blood cells with the donor’s normal red blood cells. In contrast, ascorbic acid and hyperbaric oxygen treatment are generally less effective and slower acting. These are often used in conjunction with other therapies [16].

While phenazopyridine is a recognized cause, many other medications can also induce acquired methemoglobinemia. These include local anesthetics such as benzocaine and lidocaine, sulfonamides like dapsone, antimalarials such as chloroquine, nitrates and nitrites, and antibiotics like trimethoprim-sulfamethoxazole [2,3]. Benzocaine is commonly used in topical anesthetics during endoscopic procedures, and dapsone is a frequent culprit in dermatologic and infectious disease treatment. These agents should be kept in mind when evaluating patients with unexplained cyanosis or hypoxia.

Potential causes of refractory cyanosis extend beyond toxicologic factors. Several life-threatening conditions must be ruled out during initial evaluation in the emergency setting. Differential diagnoses include but are not limited to pulmonary embolism, pneumonia, chronic obstructive pulmonary disease exacerbation, intracardiac or intrapulmonary shunts, and pulmonary effusion [19]. Airway, breathing, and circulation assessment, vital signs, and a strong history and physical exam remain critical in guiding any provider towards the next steps in further evaluation.

Limitations

Several points of weakness and limitations warrant acknowledgment. As discussed in this article, the discordant SpO₂ and ABG values should have prompted earlier measurement of the co-oximetry. Given persistent hypoxemia, the initial diagnostic approach prioritized pulmonary embolism as the leading diagnosis. A CT pulmonary angiogram was obtained, but bedside point-of-care ultrasound could have provided a rapid, noninvasive assessment for alternative causes of hypoxemia, including pneumothorax, pleural effusion, pulmonary edema, and cardiac dysfunction. Despite oxygen saturations in the 70s, the patient remained hemodynamically stable and was not tachycardic. This may have been an overlooked clue that her oxygen delivery impairment was not due to V/Q mismatch.

The patient also did not have a chest X-ray, although this is typically first-line for rapidly excluding pulmonary pathology.

The second dose of methylene blue ordered by the intensivist was given without first obtaining a repeat MetHb level. As discussed in this article, a repeat MetHb level should be obtained to guide treatment.

## Conclusions

This case highlights the importance of maintaining a broad differential diagnosis for hypoxia unresponsive to oxygen therapy, especially in patients with otherwise normal respiratory mechanics. Prompt use of co-oximetry and early recognition of causative agents are essential to initiate timely and appropriate treatment. Emergency clinicians should maintain a high suspicion for dyshemoglobinemias when evaluating for unexplained cyanosis. Methylene blue remains the standard therapy and can reverse hypoxia rapidly when administered early. Increased awareness and clinical suspicion may prevent delays in diagnosis and avoid unnecessary interventions.
